# Limited Efficacy of 3 mA Intensified tDCS of the Right Inferior Frontal Cortex for OCD Treatment: A Randomized, Double‐Blind, Sham‐Controlled Study

**DOI:** 10.1002/cns.70927

**Published:** 2026-05-24

**Authors:** Atefeh Fatehi‐Chenar, Mohsen Dadashi, Alireza Moradi, Soroush Lohrasbi, Abolfazl Ghoreishi, Arash Fazeli, Ricardo Salvador, Michael A. Nitsche, Zahra Vaziri, Mohammad Ali Salehinejad

**Affiliations:** ^1^ Department of Clinical Psychology Faculty of Medicine, Zanjan University of Medical Sciences Zanjan Iran; ^2^ Department of Clinical Psychology Kharazmi University Tehran Iran; ^3^ VESAL Rehabilitation Center Kharazmi University Karaj Iran; ^4^ Department of Psychiatry, Faculty of Medicine Zanjan University of Medical Sciences Zanjan Iran; ^5^ Brain Modeling Department Neuroelectrics Barcelona Barcelona Spain; ^6^ Department of Psychology and Neurosciences Leibniz Research Centre for Working Environment and Human Factors Dortmund Germany; ^7^ Bielefeld University, University Hospital OWL, Protestant Hospital of Bethel Foundation University Clinic of Psychiatry and Psychotherapy Bielefeld Germany; ^8^ German Center for Mental Health Bochum Germany; ^9^ School of Cognitive Sciences Institute for Research in Fundamental Sciences (IPM) Tehran Iran; ^10^ Department of Child and Adolescent Psychiatry, Psychosomatics, and Psychotherapy Medical Faculty, RWTH Aachen University Aachen Germany

**Keywords:** inferior frontal cortex, obsessive‐compulsive disorder, tDCS, transcranial direct current stimulation

## Abstract

**Objective:**

Transcranial direct current stimulation (tDCS) has been proposed for treating obsessive‐compulsive disorder (OCD) with advantages such as affordability and home‐use application. Despite a moderate therapeutic effect, previous findings have been mixed. This registered, randomized, double‐blind, sham‐controlled trial investigated the efficacy of an intensified tDCS protocol targeting the inferior frontal cortex (IFC), a relevant brain region for inhibitory control for OCD treatment.

**Methods:**

Forty patients with OCD received 20 sessions of 3 mA intensified tDCS or sham tDCS in parallel groups. The active treatment included 20 sessions of 20‐min 3 mA stimulation delivered twice per day (in 2 weeks) with 20‐min between‐session intervals. Intervention efficacy, treatment response, and cognitive functions (response inhibition, cognitive flexibility, and sustained attention) were evaluated before and after the intervention and at 2‐week, 1‐month, and 3‐month follow‐ups.

**Results:**

Blinding of patients was successful. Both groups experienced decreased OCD symptoms, anxiety, and depression. While the active tDCS group showed a significant reduction in all clinical measures from pre‐ to post‐intervention, no statistically significant between‐group differences were found. The active tDCS group showed a mean reduction of 22.38% in OCD symptoms from baseline to endpoint, with 20% (4/20) responding with ≥ 50% symptom reduction, compared to 15.25% and 0% (0/20) in the sham group. Cognitive assessments similarly revealed within‐group improvements over time, but no differences between groups.

**Conclusions:**

3 mA tDCS over the right IFC showed limited efficacy for OCD treatment, likely confounded by placebo effects, suggesting that other targeted regions may be more promising for tDCS intervention.

**Trial Registration:**

IRCT20230216057437N1

## Graphical abstract

This double‑blind RCT investigated efficacy of intensified 3 mA anodal tDCS targeting the right inferior frontal cortex for OCD treatment. Both active and sham arms improved over time, but no between‑group differences emerged on Y‑BOCS or cognition indicating limited efficacy of this intervention.

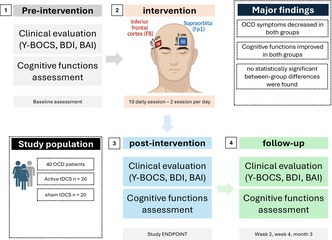

## 1 Introduction

Obsessive‐compulsive disorder (OCD) is a chronic and debilitating psychiatric condition characterized by persistent, intrusive thoughts (obsessions) and repetitive behaviors or mental acts (compulsions) performed to alleviate associated distress [1]. Affecting approximately 2%–3% of the global population, OCD imposes a significant burden on quality of life, impairing social, occupational, and personal functioning [2, 3]. The standard treatments for OCD include cognitive‐behavioral therapy (CBT), particularly exposure and response prevention, and pharmacotherapy with selective serotonin reuptake inhibitors (SSRIs). Despite their efficacy, up to 40%–60% of patients fail to achieve full symptom remission with these interventions, and many experience intolerable side effects from medications [4, 5]. This treatment gap underscores the need for novel therapeutic strategies for OCD treatment.

Transcranial direct current stimulation (tDCS) has emerged as a promising non‐invasive brain stimulation (NIBS) technique with potential applications in psychiatric and neurological disorders, including depression, anxiety, and OCD [6, 7, 8, 9, 10, 11, 12]. Unlike other forms of neuromodulation, tDCS is portable, low‐cost, and safe [13, 14], making it a promising adjunctive treatment for neuropsychiatric disorders [15]. By delivering a weak electrical current through scalp electrodes, tDCS modulates cortical excitability in targeted brain regions and affects neuroplasticity, offering a safe and tolerable adjunctive or alternative treatment option [16, 17]. A recently published comprehensive analysis of tDCS RCTs in OCD found a significant moderate therapeutic effect of tDCS specifically for upregulatory and downregulatory tDCS interventions [11]. The pathophysiology of OCD involves aberrant activity in cortico‐striato‐thalamo‐cortical (CSTC) circuits, particularly the prefrontal cortex, which regulates inhibitory control and emotional processing [18, 19, 20, 21]. Previous studies have explored tDCS as a means to alter and/or restore these circuits in OCD, with varying degrees of success [22, 23, 24].

In a recent work [25], we explored the impact of an intensified tDCS protocol at two stimulation intensities (1 and 2 mA) for OCD treatment. The intensified protocol includes 20 min of stimulation delivered twice per day with 20‐min between‐session intervals, which is able to prolong excitability enhancement [26] and induce superior therapeutic effects in previous studies [7]. We showed that modulation of the prefrontal‐supplementary motor network with intensified tDCS ameliorates clinical symptoms of OCD and results in beneficial cognitive effects [25]. This study demonstrated significant reductions in OCD symptoms in the active tDCS groups, but the 2 mA protocol, in particular, showed larger clinical and cognitive effects, indicating that higher intensities within this range may enhance therapeutic outcomes. However, the optimal stimulation parameters—intensity, electrode placement, session frequency, target region—remain under investigation [11], as subsequent studies have reported inconsistent findings, with some replicating positive effects and others finding no significant difference from sham [22, 24, 27].

Building on this foundation, the current study investigates the efficacy of an intensified tDCS protocol using 3 mA stimulation intensity for OCD treatment, given that higher currents may enhance neuromodulatory effects by more robustly altering neuronal excitability and plasticity [28]. In contrast to our previous work, which targeted the right pre‐supplementary motor area (pre‐SMA) and left dorsolateral prefrontal cortex (DLPFC) with a 1 and 2 mA current [25], this RCT employs a 3 mA current with the anode placed over the right inferior frontal cortex (IFC) and the cathode over the left supraorbital area. This stimulation montage targets the right IFC, a region involved in response inhibition [29, 30]—a core deficit in OCD—and its modulation with anodal stimulation can facilitate response inhibition through dynamic modulation of the fronto‐basal ganglia network [31]. However, OCD is a clinically and neurobiologically heterogeneous disorder, and symptom dimensions and intrusive thoughts map onto at least partly distinct neurobiological mechanisms rather than reflecting a fully unitary syndrome [3, 32]. Neuroimaging studies further suggest that these symptom dimensions are associated with both overlapping and partially distinct abnormalities in cortico‐striato‐thalamo‐cortical and prefrontal networks [30, 33]. Accordingly, the right IFC may not be an equally optimal target region across all OCD symptom profiles.

In this registered clinical trial (Trial ID: IRCT20230216057437N1), we investigated the impact of intensified tDCS at 3 mA intensity over the right IFC on symptom reduction and cognitive deficits in patients with OCD. The primary objectives of this study are: (1) to evaluate the efficacy of 3 mA intensified tDCS of the right IFC in reducing OCD symptoms, as measured by the Yale‐Brown Obsessive‐Compulsive Scale, compared to sham stimulation in a double‐blind RCT; (2) to assess its effects on secondary outcomes, including anxiety, depression, and cognitive functions; and (3) to evaluate whether and how the right IFC is a promising stimulation target for OCD treatment given the previous mixed results about targeting the pre‐supplementary motor area and lateral prefrontal cortex. The novel aspects of this work include the application of 3 mA stimulation in an intensified protocol and targeting the right IFC for OCD treatment.

## 2 Methods

### 2.1 Participants

**FIGURE 1:**
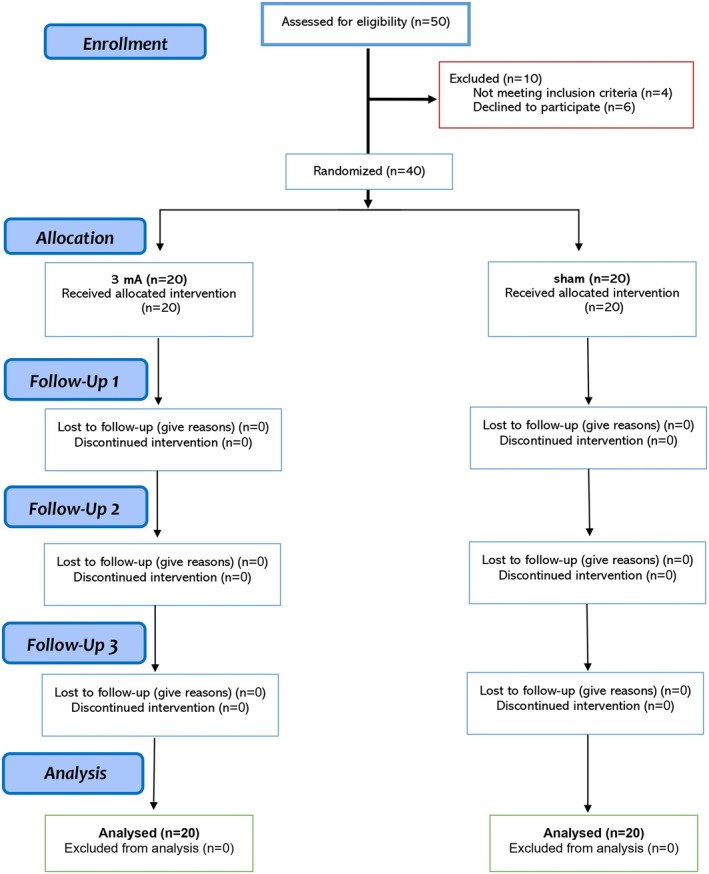
CONSORT flow diagram of study inclusion.

This study was a randomized, double‐blind, sham‐controlled trial with a parallel‐group design to prevent blinding failure and carry‐over effects. Forty individuals diagnosed with OCD (mean age = 35.25, SD = 10.56, 23 females) were recruited from several neuropsychiatric clinics in Zanjan, Iran, from April 2023 to August 2024. Patients were randomly assigned to the active (*N* = 20) or sham (*N* = 20) stimulation groups using the block randomization method (block size of 4). Sample size was calculated a priori based on a medium effect size suggested for tDCS studies (*f* = 0.25, *α* = 0.05, power = 0.95, *N* = 32, mixed‐model ANOVA with 5 measurements). We added 8 more subjects to the total sample size to compensate for potential dropouts, yet none of the participants dropped out before completing the study (Figure 1). The inclusion criteria were: (1) diagnosis of OCD according to Structured Clinical Interview for DSM‐5 (SCID‐5) [34], (2) 18–50 years old, (3) non‐smoker, (4) no previous history of neurological diseases, brain surgery, epilepsy, seizures, brain damage, head injury, or metal brain implants, and (5) absence of other psychiatric diagnoses. Clinically relevant depressive and anxiety symptoms were not exclusionary, given their high prevalence in OCD, and were assessed dimensionally using the BDI‐II and BAI. Those patients on medication from active (*N = 6*) and sham (*N* = 5) groups were receiving stable doses for at least 6 weeks before the experiment, up to the end of the 3‐month follow‐up. All participants were native speakers and had normal or corrected‐to‐normal vision. This was a registered clinical trial (Registration ID: IRCT20230216057437N1) approved by the Ethics Committee of the Zanjan University of Medical Sciences (Ethics code: IR.ZUMS.REC.1401.339). Participants gave their written informed consent before participation (see Table 1 for demographics).

**TABLE 1:** Demographic data.

		Active tDCS	Sham tDCS	*p* *
Sample size (*n*)		20	20	
Age – Mean (SD)		34.55 (10.67)	35.95 (10.68)	0.681
Sex – Male (female)		10 (10)	7 (13)	0.337
Marital Status (*n*)	Single	10	8	0.925
	Married	7	9	
	Divorced	2	2	
	Widowed	1	1	
Education (*n*)	Under diploma	4	1	0.821
	Diploma	4	8	
	Undergraduate	4	2	
	Bachelor's degree	8	7	
	Master's degree	0	2	
Occupation (*n*)	Student	4	1	0.468
	Employee	3	2	
	Self‐employed	4	5	
	Unemployed	9	12	
On medication (*n*)	Yes	6	5	0.723
	No	14	15	
Duration of disease – Mean (SD)		9.25 (8.10)	9.55 (8.46)	0.887
Depressive/anxiety symptoms (*n*)		8	14	0.425

Abbreviations: M, Mean; SD, Standard deviation.

* Between‐group differences in demographic variables were explored by Chi‐square tests or Fisher's exact test for categorical variables and F‐tests for continuous variables.

### 2.2 Outcome Measures

#### 2.2.1 Clinical Measures

The primary outcome was change in OCD symptom severity, as measured by the Yale‐Brown Obsessive‐Compulsive Scale (Y‐BOCS) [35], at the prespecified primary endpoint immediately after completion of the intervention (i.e., after the 20th stimulation session; post‐intervention). Additionally, anxiety and depressive states were tested by the Beck Anxiety Inventory (BAI) [36] and the Beck Depression Inventory (BDI‐II) [37], respectively. Detailed descriptions of measures are in the supplementary content.

#### 2.2.2 Cognitive Performance

The Cambridge Neuropsychological Test Automated Battery (CANTAB) is a computerized collection of cognitive assessments that were used in this study to assess the following areas: cognitive flexibility (assessed through the Intra‐Extra Dimensional Set Shift task), response inhibition (evaluated via the Stop Signal Task), and sustained attention (assessed using the Rapid Visual Information Processing task). The CANTAB tests have been thoroughly detailed elsewhere [38, 39]. Detailed descriptions of measures are in the supplementary content.

### 2.3 tDCS

**FIGURE 2:**
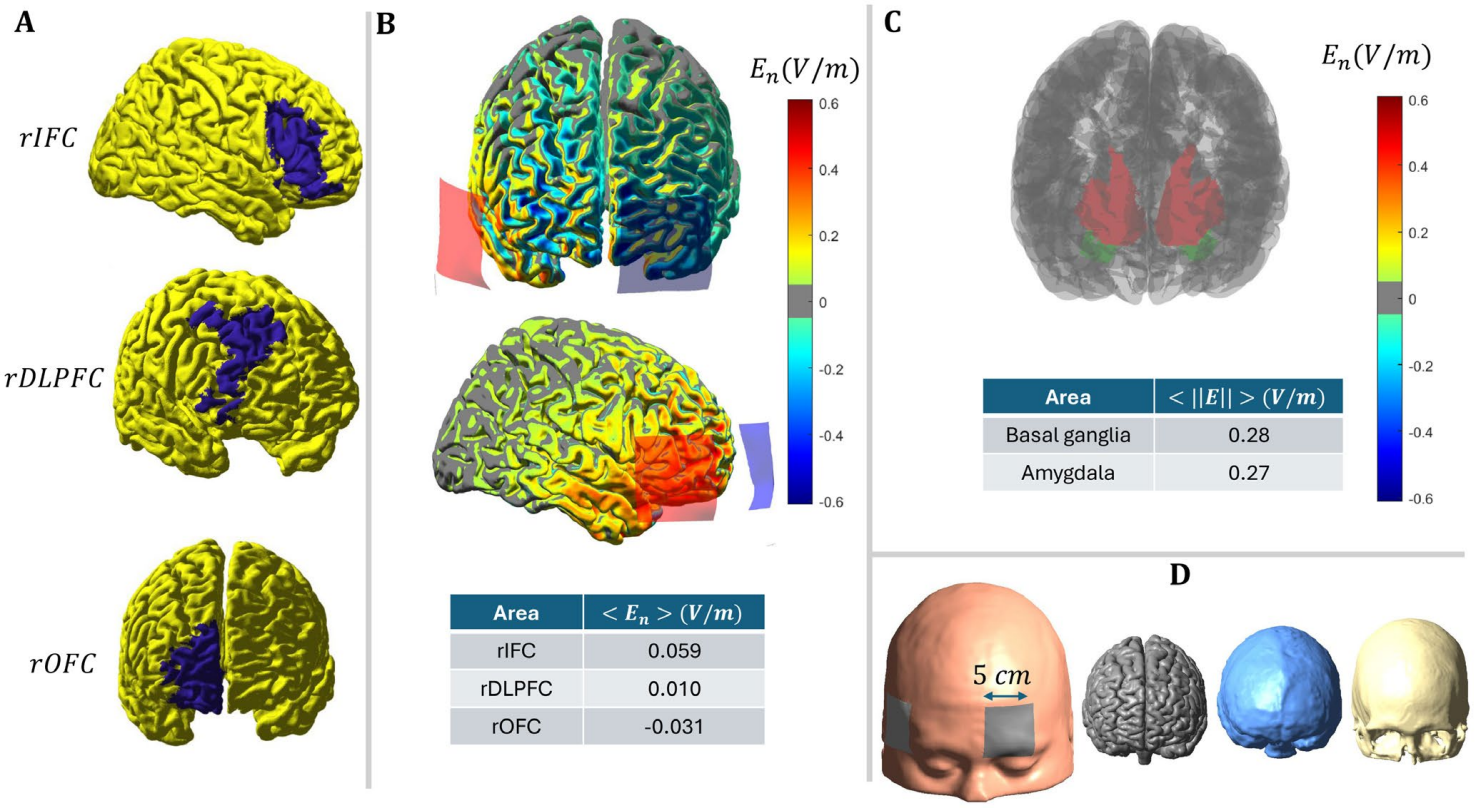
(A) The location of the right IFC/rIFG [BA 44, 45, 47], right DLPFC [BA 9, 46], and right OFC [BA 10–14] in the brain. (B) Distribution of the induced electrical field (E‐field) with the anode placed over each target region and the cathode positioned on the left supraorbital area, using a current intensity of 3 mA. (C) The average magnitude of the E‐field was calculated in subcortical areas, including the basal ganglia and amygdala. (D) Volume conduction and visualization of anodal and cathodal electrodes placed over the right IFC (F8) and the left supraorbital area (Fp1).

Direct currents were generated by an electrical stimulator (MindAlive, Canada) applied through a pair of saline‐soaked sponge electrodes (5 × 5 cm) delivered for 20 min on 10 consecutive days (2 sessions per day with a 20 min interval, 20 sessions in total). With 5 × 5 cm sponge electrodes, the applied current density was approximately 0.12 mA/cm^2^, and each 20‐min session delivered 3.6 C of charge. These parameters are within conventional human tDCS safety limits reported in the literature (≤ 4 mA, ≤ 40 min, ≤ 7.2 C per session), although higher current intensity is expected to increase transient scalp sensations [13]. In both active (3‐mA) and sham conditions, anodal and cathodal electrodes were placed over the F8 (rIFC) and Fp1 (left supraorbital/frontopolar), respectively, to guarantee a minimum 6 cm distance between the edges of the electrodes [40] (Figure 2). The protocol was adapted from a study demonstrating that anodal tDCS over the right inferior frontal cortex enhances response inhibition by altering functional connectivity within the fronto‐basal ganglia inhibitory network [31], which is involved in OCD pathophysiology [30]. In sham stimulation, the electrical current was ramped up and down for 30 s each to generate the same sensation as in the active condition and then turned off [41]. A side‐effect survey was done after each tDCS session [42]. To guarantee blinding, tDCS was applied by independent investigators who were not involved in outcome measures rating [43]. Blinding efficacy was explored among patients at the end of the last stimulation session by asking participants whether they received a real or a non‐real stimulation. After finalizing the study protocol, the patients in the sham group were assigned to active tDCS intervention, but the latter procedure was beyond the focus of the study protocol. The results of electrical field modeling are presented in Figure 2, illustrating the average induced electrical field in the primary target region (r‐IFC) compared to nearby areas such as the right OFC and right DLPFC, as well as subcortical regions relevant to OCD pathophysiology.

### 2.4 Procedure

**FIGURE 3:**
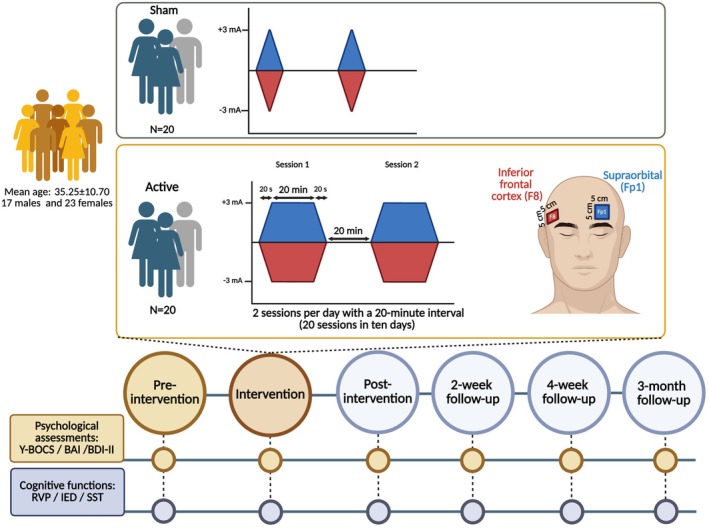
Experimental procedure. Forty patients with obsessive–compulsive disorder were randomized to active 3 mA tDCS or sham stimulation (*n* = 20 per group). Active stimulation was delivered over 10 consecutive days as two 20‐min sessions per day, separated by a 20‐min interval, with 20‐s ramp‐up and ramp‐down periods. The anode was positioned over the right inferior frontal cortex/inferior frontal gyrus (F8), and the cathode over the left supraorbital area (Fp1), using 5 × 5 cm electrodes. Sham stimulation used the same montage but included only brief ramp‐up/ramp‐down periods. Clinical outcomes (Y‐BOCS, BAI, BDI‐II) and cognitive functions (RVP, IED, SST) were assessed at pre‐intervention, post‐intervention, and at 2‐week, 4‐week, and 3‐month follow‐ups. BAI, Beck Anxiety Inventory; BDI‐II, Beck Depression Inventory‐II; IED, Intra–Extra Dimensional Set Shift; RVP, Rapid Visual Information Processing; SST, Stop Signal Task; tDCS, transcranial direct current stimulation; Y‐BOCS, Yale–Brown Obsessive–Compulsive Scale.

Prior to the experiment, participants completed a brief questionnaire to evaluate their suitability for brain stimulation. All participants received 20 sessions of tDCS in 2 weeks. All stimulation sessions took place between 11:00 and 14:00, and participants were not under sleep pressure [44, 45]. Clinical and cognitive measures were evaluated using the same assessment protocols before the first intervention (pre‐intervention), immediately after the end of the last intervention (post‐intervention), and also at 2‐week, 4‐week, and 3‐month intervals following the last stimulation session (follow‐ups). Patients were instructed about the tasks before the beginning of the experiment, and the task stimuli order was randomized in each measurement. After each stimulation session, participants in both groups rated tDCS‐related side effects (itching, burning, pain, skin redness, and trouble concentrating) on a Likert‐type scale [42]. None of the patients received psychotherapy during the study. Participants were blind to the study hypotheses and stimulation conditions. The experimenter who conducted the outcome measures was blinded to the tDCS conditions (Figure 3).

### 2.5 Statistical Analysis

Data were analyzed with the statistical package SPSS, version 27.0 (IBM, SPSS Inc., Chicago, IL) and GraphPad Prism 9.1 (GraphPad Software, San Diego, California). The normality and homogeneity of data variance were confirmed by Shapiro–Wilk and Levene's tests, respectively. Mixed model ANOVAs were conducted for the dependent variables (clinical measures: obsessive‐compulsive, depression, and anxiety symptoms; cognitive measures: sustained attention, cognitive flexibility, response inhibition) with “group” (active vs. sham) as the between‐subject factor and time (pre‐intervention, post‐intervention, 2‐week, 4‐week, and 3‐month follow‐ups) as the within‐subject factor. Mauchly's test was used to assess sphericity for the repeated‐measures factor. When sphericity was violated (*p* < 0.05), degrees of freedom were corrected using the Greenhouse–Geisser method. Corrected degrees of freedom are reported in the Results where applicable. When sphericity assumptions were met, uncorrected degrees of freedom are reported. In the event of significant findings in the ANOVAs, LSD post hoc comparisons were executed across time points for each group and between groups for each time point. Average side‐effect ratings were calculated for each participant and compared between groups using one‐way ANOVAs for each side‐effect category. The association between OCD subtypes and symptom reduction was investigated using Pearson's correlational analysis. Blinding efficacy was examined with the chi‐squared test for Independence on participants' guesses of blinding (0, 1) and Bang's Blinding Index (−1, 1) [46], with negative values suggesting that participants frequently guessed the opposite of their actual treatment. The critical level of significance was 0.05 for all statistical analyses.

## 3 Results

### 3.1 Baseline Assessment, Safety Outcomes, and Blinding Efficacy

Table 1 summarizes demographic data, with no significant differences observed across groups. Tables S1 and S2 show the mean and standard deviation of outcome measures (overall and symptom category), with no significant between‐group differences in baseline measurements of outcome variables. Table S3 summarizes the average reported side‐effect ratings across sessions for each group. Side effects were monitored after every tDCS session in both groups using a Likert‐type scale. The results revealed significantly higher ratings of itching (*F*_1,38_ = 20.10, *p* < 0.001), burning (*F*_1,38_ = 47.53, *p* < 0.001), pain (*F*_1,38_ = 12.36, *p* = 0.001), and skin redness (*F*_1,38_ = 28.21, *p* < 0.001) in the active tDCS group vs. the sham group. No significant difference was found in trouble concentrating between the two groups (*F*_(1,38)_ = 0.114, *p* = 0.738) (Table S3). Regarding blinding efficacy, 3 out of 20 participants (15%) in the active group noticed their condition correctly, while 17 out of 20 (85%) did not. In the sham group, 4 out of 20 participants (20%) noticed their condition correctly, whereas 16 out of 20 (80%) did not. The results of the Chi‐Square test (χ2 = 0.173; *p* = 0.677) and Bang's Blinding Index (active = −0.7; sham = −0.6) indicated that the blinding was successful, suggesting that participants' awareness of their tDCS condition did not significantly influence their perception of side effects.

### 3.2 Clinical Outcome Measures

#### 3.2.1 OCD Symptoms

**FIGURE 4:**
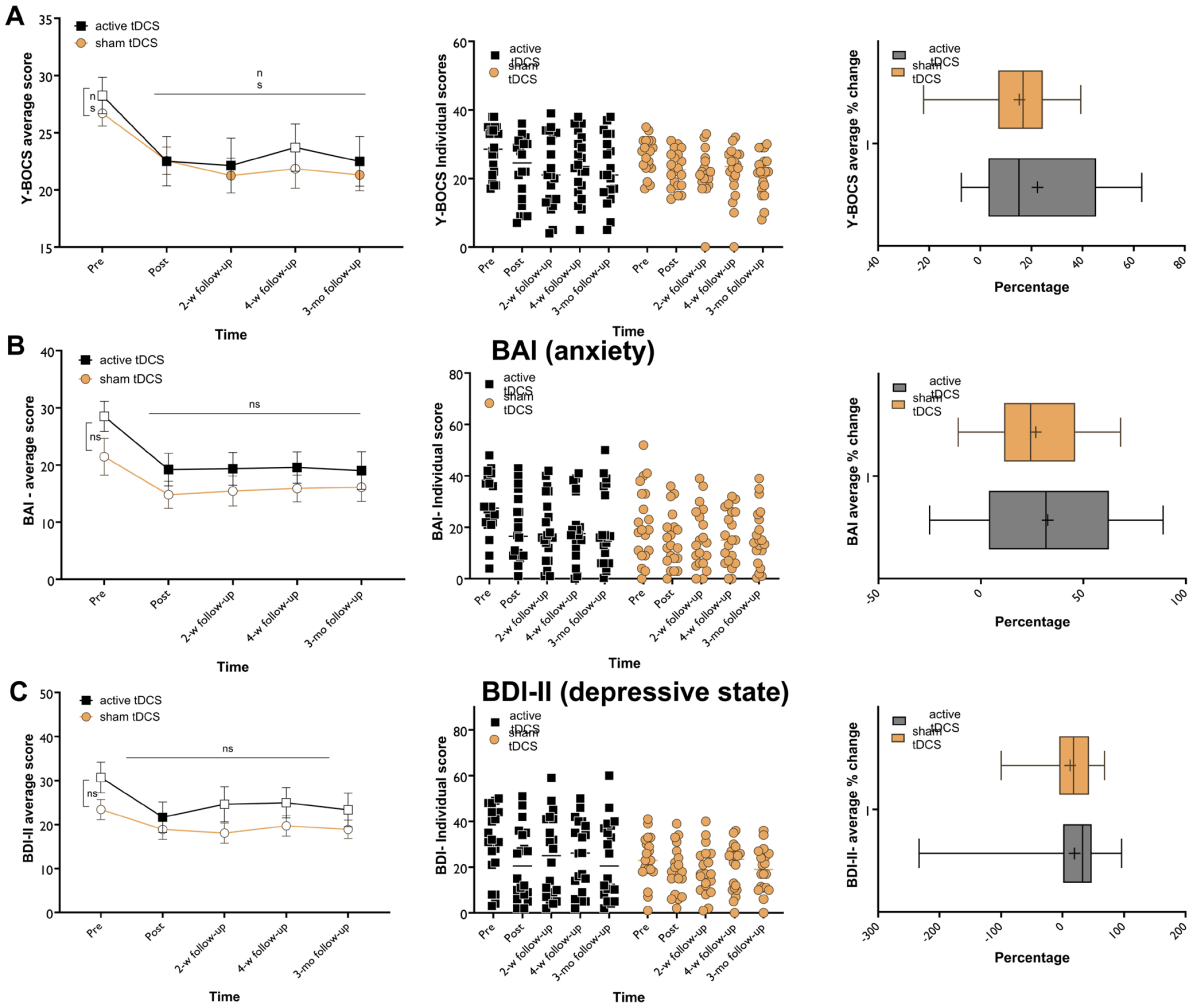
OCD symptoms, depression, and anxiety states were measured by the Y‐BOCS (A), BAI (B), and BDI‐II (C) before, after, and up to 2 weeks, 1 month, and 3 months following the intervention. In the left panel, filled symbols indicate significant differences at each time point compared to pre‐intervention scores. The middle panel displays a scatter plot of clinical scores for each group across time points. The right panel shows the mean score change from the baseline to the study endpoint (week 2 after the 20_th_ session) for the respective measure. The horizontal bar shows the median, the + shows the mean, the upper and lower boundaries show the 25th and 75th percentiles, respectively, and the whiskers show the 1–99th percentile. Pairwise comparisons were conducted with post hoc LSD tests, and error bars indicate the standard error of the mean (s.e.m.). BAI, beck anxiety inventory; BDI‐II, beck depression inventory‐II; mo, month; ns, non‐significant; tDCS, transcranial direct current stimulation; w, week; Y‐BOCS, yale‐brown obsessive‐compulsive scale.

For Y‐BOCS scores, a significant main effect of time (*F*_3.276,124.470_ = 13.884, *p* < 0.001, *ηp* ^*2*^ = 0.268) but no main effect of group (*F*_1,38_ = 0.234, *p* = 0.632, *ηp* ^*2*^ = 0.006) or the time×group interaction (*F*_3.276,124.470_ = 0.326, *p* = 0.823, *ηp* ^*2*^ = 0.009) was observed. Within‐group post hoc LSD comparisons indicated significant Y‐BOCS score reductions from baseline in the active‐tDCS group at post‐intervention (*p* = 0.023), 2‐week (*p* = 0.015), and 3‐month follow‐ups (*p* = 0.023). The sham‐tDCS group showed significant reductions only at the 2‐week (*p* = 0.031) and 3‐month follow‐ups (*p* = 0.033). No significant between‐group differences were found at any time point, including the baseline differences (Figure 4A).

#### 3.2.2 Anxiety State

For the BAI scores, a significant main effect of time (*F*_4,152_ = 13.221, *p* < 0.001, *ηp* ^*2*^ = 0.258) and no effect of group (*F*_1,38_ = 1.542, *p* = 0.222, *ηp* ^2^ = 0.039) and time × group interaction (*F*_4,152_ = 0.728, *p* = 0.574, *ηp* ^2^ = 0.019) were observed. Post hoc LSD comparisons indicated a significant decrease in BAI scores only in the active‐tDCS group at post‐intervention *(p* = 0.018), 2‐week *(p* = 0.020), 4‐week *(p* = 0.023), and at 3‐month (*p* = 0.016) follow‐ups vs. pre‐intervention. Regarding between‐group analysis, no significant differences were observed at any time point (Figure 4B).

#### 3.2.3 Depressive State

BDI scores showed a significant main effect of time (*F*_4,152_ = 6.635, *p* < 0.001, *ηp* ^*2*^ = 0.149) and no main effect of group (*F*_1,38_ = 1.854, *p* = 0.181, *η*p^2^ = 0.047) or time×group interaction (*F*_4,152_ = 0.734, *p* = 0.570, *η*p^2^ = 0.019). Within‐group post hoc LSD comparisons showed a significant reduction in BDI scores only in the active‐tDCS group at post‐intervention *(p* = 0.036) compared to the pre‐intervention. Between‐group analysis revealed no significant differences between the active and sham tDCS groups at any time point, including the baseline differences (Figure 4C).

#### 3.2.4 Symptom Reduction and Treatment Response

We also investigated the average symptom reduction treatment response (defined as at least 50% symptom reduction after the intervention) across groups. For OCD symptoms, the active tDCS group showed an average reduction of 22.38%, with 20% of patients responding, compared to a 15.25% symptom reduction and 0% response in the sham group. For BAI scores, active tDCS led to a 32.58% average reduction and a 40% response rate, while the sham group showed a reduction of 26.73% and a 20% response rate, suggesting a partial placebo effect. For depressive symptoms, active tDCS and sham groups had average reductions of 19.33% and 12.42%, respectively, with response rates of 20% and 15%, respectively, suggesting a partial placebo effect. Exploratory correlational analyses did not reveal significant associations between OCD symptom dimensions/subtypes and treatment response. However, these analyses should be interpreted cautiously because the study was not powered to detect symptom‐dimension‐specific effects in a heterogeneous disorder such as OCD.

### 3.3 Cognitive Outcome Measures

#### 3.3.1 The Rapid Visual Information Processing (RVP)

**FIGURE 5:**
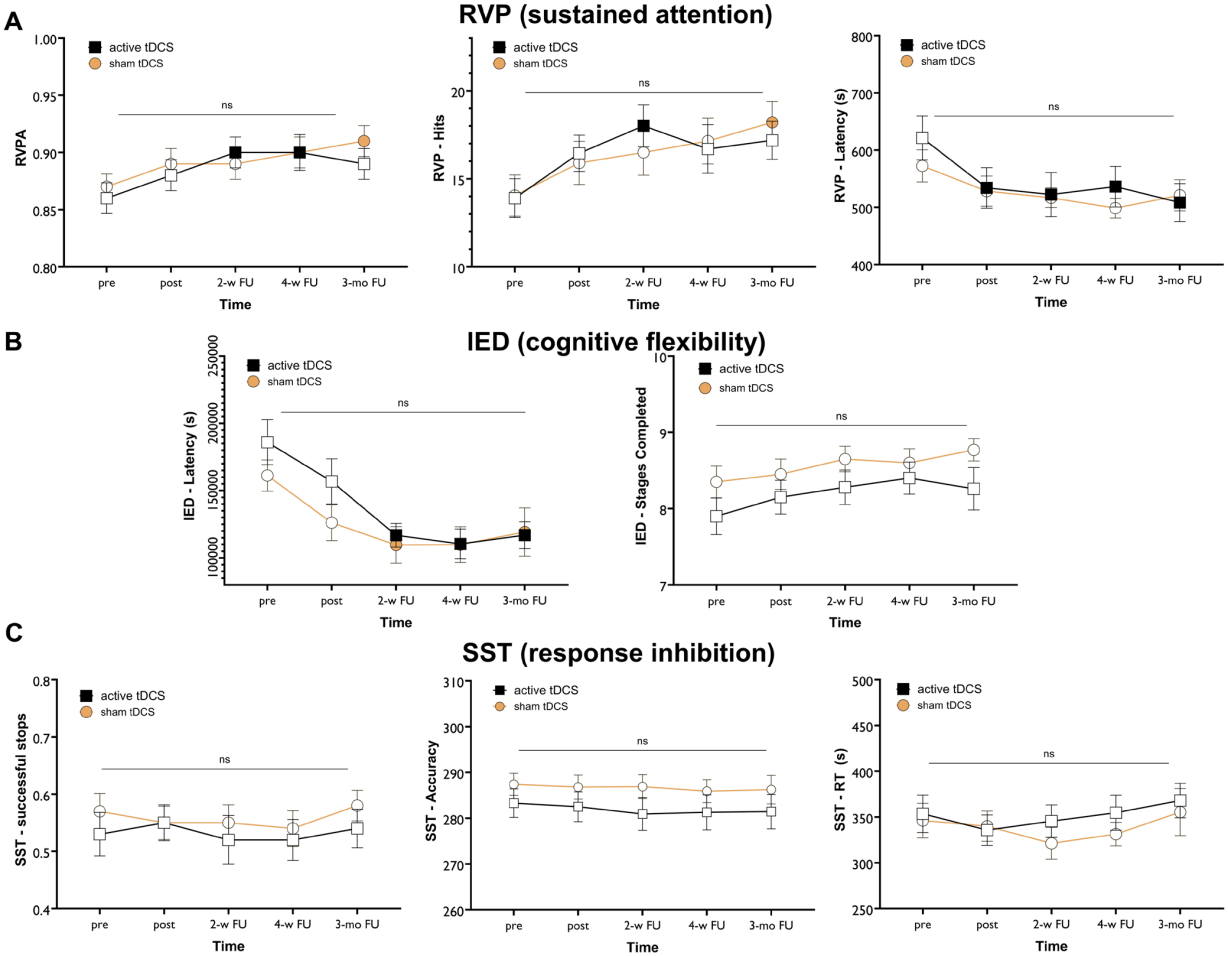
Cognitive performance was evaluated before and after the last session of 3 mA intensified and sham tDCS using the CANTAB neuropsychological test battery for OCD, which includes measures of sustained attention (A), cognitive flexibility (B), and response inhibition (C). Filled symbols indicate significant differences at each time point compared to pre‐intervention scores. Post hoc tests were conducted with LSD pairwise comparisons, and error bars indicate the standard error of the mean (s.e.m.). FU, follow‐up; IED, intra‐extra dimensional set shift; mo, month; ns, non‐significant; RVP, rapid visual processing; s, second; SST, stop signal task; tDCS, transcranial direct current stimulation; w, week.

Sustained attention was assessed with the RVP with RVP‐A (accuracy of target detection adjusted for false positives), hits (accuracy), and latency of correct responses as major outcome measures. The mixed‐model ANOVA revealed a significant main effect of time on RVP‐A scores (*F*_4,152_ = 9.462, *p* < 0.001, *ηp* ^2^ = 0.199), but no significant main effect of group (*F*_1,38_ = 0.148, *p* = 0.702, *ηp* ^2^ = 0.004) or time×group interaction (*F*_4,152_ = 1.025, *p* = 0.396, *ηp* ^2^ = 0.026). Post hoc analyses showed significantly higher RVP‐A scores in the active tDCS group at the 2‐week (*p* = 0.020) and 4‐week (*p* = 0.046) follow‐ups compared to pre‐intervention, and in the sham tDCS group at the 3‐month follow‐up (*p* = 0.032). Analysis of accuracy (hits) similarly showed a significant main effect of time (*F*_4,152_ = 9.160, *p* < 0.001, *ηp* ^2^ = 0.194) with non‐significant group (*F*_1,38_ = 0.004, *p* = 0.952, *ηp* ^2^ = 0.000) and interaction effects (*F*_4,152_ = 1.010, *p* = 0.404, *ηp* ^2^ = 0.026). Hits significantly increased in the active‐tDCS group at the 2‐week follow‐up (*p* = 0.016) and in the sham tDCS group at the 3‐month follow‐up (*p* = 0.015) relative to pre‐intervention. For latency, there was also a significant main effect of time (*F*_4,120_ = 6.925, *p* < 0.001, *ηp* ^2^ = 0.188), while group (*F*_1,30_ = 0.205, *p* = 0.654, *ηp* ^2^ = 0.007) and interaction effects (*F*_4,120_ = 0.715, *p* = 0.583, *ηp* ^2^ = 0.023) were not significant. Post hoc tests revealed significantly reduced latency in the active tDCS group at post‐intervention (*p* = 0.041), 2‐week (*p* = 0.036), 4‐week (*p* = 0.032), and 3‐month follow‐ups (*p* = 0.001) compared to pre‐intervention. No significant latency changes were observed in the sham tDCS group. No significant between‐group differences were found for RVP‐A, hits, or latency (Figure 5A).

#### 3.3.2 Intra‐Extra Dimensional Set Shift (IED)

Cognitive flexibility was measured with the IED. The ANOVA revealed a significant main effect of time for latency (*F*_3.032,115.214_ = 16.531, *p* < 0.001, *ηp* ^2^ = 0.303) and stages completed (*F*_4,152_ = 3.366, *p* = 0.011, *ηp* ^2^ = 0.081). Post hoc analyses revealed significant latency reductions from baseline in both the active‐tDCS group (2‐week: *p* = 0.0004; 4‐week: *p* = 0.0001; 3‐month: *p* = 0.0004) and the sham‐tDCS group (2‐week: *p* = 0.008; 4‐week: *p* = 0.008; 3‐month: *p* = 0.030). Significant effects for stages completed did not survive pairwise comparison. The effect of group was not significant for latency (*F*_(1,38)_ = 0.633, *p* = 0.431, *ηp* ^2^ = 0.016) and stages completed (*F*_(1,38)_ = 2.132, *p* = 0.152, *ηp* ^2^ = 0.053). The time×group interaction also showed no significant effects on total latency (*F*_3.03,115.21_ = 1.268, *p* = 0.289, *ηp* ^2^ = 0.032), and stages completed (*F*_4,152_ = 0.435, *p* = 0.783, *ηp* ^2^ = 0.011) (Figure 5B).

#### 3.3.3 Stop Signal Task (SST)

Response inhibition was measured by the SST. The ANOVA revealed no significant main effects of time or group, nor a significant time × group interaction, for any outcome measure (accuracy, reaction time, or proportion of successful stops). The detailed statistical values are as follows: Time—accuracy (*F*_2.70,102.90_ = 0.399, *p* = 0.733, *ηp* ^2^ = 0.010), reaction time (*F*_2.60,98.82_ = 1.362, *p* = 0.261, *ηp* ^2^ = 0.035), and proportion of successful stops (*F*_4,152_ = 0.447, *p* = 0.774, *ηp* ^2^ = 0.012). Main effect of group: accuracy (*F*_1,38_ = 1.473, *p* = 0.232, *ηp* ^2^ = 0.037), reaction time (*F*_1,38_ = 0.394, *p* = 0.534, *ηp* ^2^ = 0.010), and proportion of successful stops (*F*_1,38_ = 0.563, *p* = 0.458, *ηp* ^2^ = 0.015). time × group interaction: accuracy (*F*_2.708,102.904_ = 0.103, *p* = 0.947, *ηp* ^2^ = 0.003), reaction time (*F*_2.601,98.820_ = 0.390, *p* = 0.732, *ηp* ^2^ = 0.010), and proportion of successful stops (*F*_4,152_ = 0.143, *p* = 0.966, *ηp* ^2^ = 0.004) (Figure 5C).

## 4 Discussion

This randomized, double‐blind, sham‐controlled trial investigated the efficacy of 3 mA intensified tDCS over the right IFC for treating OCD in 40 patients. The primary finding was a significant reduction in OCD symptoms (Y‐BOCS scores), anxiety (BAI scores), and depression (BDI‐II scores) over time in both the active and sham tDCS groups. Despite a significant post vs. pre‐intervention reduction of all clinical measures only in the active tDCS group, and a higher number of responders in the active group, no statistically significant differences emerged between the groups (active vs. sham) across clinical outcome measures. Similarly, cognitive assessments of sustained attention, cognitive flexibility, and response inhibition showed within‐group improvements over time but no group‐specific effects. These results suggest that the intensified 3 mA anodal tDCS over the right IFC with cathodal stimulation over the left supraorbital area did not provide a therapeutic advantage beyond placebo effects. In what follows, we first discuss the results with respect to the target region and stimulation intensity, two novel aspects of the current study. We then discuss the observed placebo effects and potential reasons, and finally compare the current 3 mA intensified tDCS protocol with the current tDCS literature in OCD.

### 4.1 Limited Efficacy: Relevance of Target Region and Stimulation Intensity

#### 4.1.1 Is the Right IFC a Reliable Target Region for OCD Treatment?

One novel aspect of our tDCS protocol was targeting the right IFC with anodal stimulation based on previous work showing its central role in response inhibition [29], and specifically a study that showed anodal right IFC tDCS facilitates response inhibition and functional connectivity between the right pre‐SMA and subthalamic nuclei [31], two highly involved regions in OCD pathophysiology [21, 30]. This rationale is relevant to OCD because inhibitory‐control deficits and altered neural recruitment during inhibitory control have repeatedly been implicated in the disorder [19, 30]. At the same time, OCD is a heterogeneous condition in which different symptom dimensions may rely on partially distinct neural systems, despite some shared CSTC involvement [32, 33]. Therefore, although the right IFC is a theoretically plausible target for inhibitory‐control‐related aspects of OCD, it may not be equally suitable across all symptom profiles. More specifically, the right IFC may be more relevant for symptom expression linked to compulsive responding or broader deficits in cognitive control, whereas other symptom dimensions may depend more strongly on orbitofrontal, anterior cingulate, pre‐SMA/SMA, insular, or other frontostriatal circuit dysfunction [21, 47, 48].

Additionally, the orbitofrontal cortex (OFC) is anatomically very close to the right IFC and is hyperactive in OCD patients [21]. It is possible that the 3 mA anodal stimulation, with its diffuse induced electrical field, has also increased excitability in the OFC or failed to sufficiently regulate hyperactivity in the cortico‐striato‐thalamo‐cortical circuit, which contributes to OCD thoughts and behavior [21]. Conversely, cathodal stimulation of the left frontopolar region, which was aimed to inhibit the orbitofrontal cortex, might decrease excitability in the DLPFC, as it is anatomically continuous with and immediately anterior to the DLPFC. In this respect, a recent functional neuroimaging study of executive function showed that OCD patients show moderate to very strong evidence of weaker activation of the DLPFC, precuneus, frontal eye fields, and inferior parietal lobule during executive functioning tasks as compared to healthy controls [49]. The expected modulatory effect of cathodal stimulation over the left frontopolar region should not be ignored. Although inhibiting the left frontopolar was anticipated, cathodal tDCS at higher intensities may not achieve this due to the complexities involved [50]. These heterogeneities may partly explain the limited efficacy observed in the present study, and future trials should therefore consider stratified randomization, symptom‐dimension‐based subgroup analyses, or individualized target selection based on dominant symptom dimensions and circuit‐level abnormalities. A priori modeled or computationally optimized tDCS interventions [51, 52] for determining effective interventions are also critical for effective modulation of target regions.

#### 4.1.2 3 mA tDCS Could Be Too Much

The limited efficacy of 3 mA tDCS may also relate to the non‐linear nature of tDCS dose–response effects. The rationale for selecting 3 mA in the present study was based on our previous OCD trial showing larger and more convergent clinical benefits with 2 mA than with 1 mA in an intensified protocol [25]. However, this does not imply that efficacy should continue to increase monotonically at higher intensities. Because the current study did not include an active 2 mA comparator arm, it cannot isolate whether the limited efficacy observed here was attributable specifically to the 3 mA intensity, to the different stimulation target/montage, or to the interaction of both factors. Indeed, it is possible that the 3 mA tDCS over the left frontopolar cortex and right IFC may have induced counterproductive effects. In a physiological study of anodal tDCS over the motor cortex, stimulation intensities between 1 and 3 mA all increased cortical excitability, but 3 mA was not clearly superior to lower intensities [28]. Moreover, in a subsequent study, the same intervention (3 mA anodal tDCS with 20 min interval) was conducted on motor cortical excitability, and the findings showed that, in contrast to 1 mA tDCS, which prolonged after‐effects for 24 h, the 3 mA tDCS after‐effects were limited to 120 min [53]. These findings suggest that raising intensity may not necessarily strengthen the desired plastic effects and may, under some conditions, even reduce the efficacy of spaced stimulation protocols. Accordingly, it is possible that 3 mA stimulation in the present intensified protocol exceeded the optimal range for inducing sustained therapeutic plasticity.

A hypothesized mechanism here is calcium overflow as a result of 3 mA stimulation. While moderate increases in intracellular calcium facilitate long‐term potentiation (LTP), excessive calcium influx can trigger depotentiation or long‐term depression (LTD), preventing further plasticity induction [54, 55]. It is possible that intensified tDCS of the prefrontal regions, as shown in the motor cortex [53], has a stronger activating effect on NMDAR, and the resulting higher amount of calcium influx induces counterbalancing effects with a second stimulation [56]. Furthermore, we applied the 3 mA tDCS in intensified mode (20 min stimulation + 20 min rest + 20 min stimulation), which was shown to limit later LTP compared to 1 mA [53]. The second stimulation period was during the aftereffects of the first period, which could prolong excitability in previous tDCS studies in motor excitability only at 1 mA but not 3 mA intensity [26, 53, 57]. While in our previous work, we showed a significantly larger therapeutic efficacy of the 2 mA intensified tDCS vs. 1 mA [25], the 3 mA intensity might have been excessive. This comparison is, however, limited by the different target regions used in our previous study (left DLPFC and right pre‐SMA).

### 4.2 The Placebo Effect

The observed improvements in both active and sham groups indicate a placebo effect, consistent with prior NIBS studies. A meta‐analysis of rTMS trials in depression found a large placebo response linked to depression improvement [58]. In tDCS studies, similar antidepressant effects were reported [59]. In treatment‐resistant OCD, two recent studies found that active tDCS was not superior to sham despite symptom reduction [22, 60]. In one study, 20 daily sessions of cathodal SMA tDCS significantly reduced OCD symptoms compared to sham, but no between‐group differences were found in treatment response and anxiety/depression symptom reduction [60]. The other study with 10 sessions of 2 mA tDCS (cathodal SMA‐anodal Fp2) reported a significant Y‐BOCS score decrease over time in both groups [22]. These findings parallel our study, where the sham group's 15.27% reduction in Y‐BOCS scores and 26.73% reduction in BAI scores suggest the presence of a placebo response.

The placebo effect is influenced by multiple factors, including patients' belief in the treatment, trust in healthcare providers, ritualistic treatment procedures, and the therapeutic context, particularly for those with prior unsuccessful treatments. Patients' expectations of improvement may alter neurophysiology and emotional states [61], which may contribute to OCD symptom reduction. Clinician‐rated scales show a stronger placebo effect than self‐reported measures [62]. Furthermore, NIBS techniques, like tDCS and rTMS, may produce greater placebo effects compared to other treatments [63]. Lastly, a meta‐analysis of OCD RCTs (regardless of intervention type) showed that placebos are about 50% as effective as active interventions in OCD trials, especially in younger patients [62]. Our results also highlight these factors, indicating that placebo effects may have been underestimated in prior tDCS studies for OCD. That said, the variability of tDCS outcomes may also partly reflect state‐dependent neuroplasticity, which can affect anodal tDCS‐induced LTP‐like effects via NMDA‐related mechanisms [64, 65].

### 4.3 Comparison With Existing tDCS Studies in OCD

Previous tDCS studies in OCD have mostly used 1–2 mA stimulation intensity over other regions (e.g., dorsolateral prefrontal cortex, pre‐SMA) [11]. Some studies reported modest symptom reductions, mostly with cathodal pre‐SMA and anodal left DLPFC tDCS [24, 25, 27], while others found no difference from sham [22, 60]. Nonetheless, a recent comprehensive analysis of tDCS RCTs in OCD found a moderate therapeutic effect of tDCS for OCD treatment with specific stimulation protocols (e.g., cathodal pre‐SMA/OFC, anodal lateral PFC tDCS) or parameters (twice stimulation daily, 2 mA intensity) demonstrating significant clinical efficacy [11]. The current study's use of 3 mA extends this exploration but suggests that this specific tDCS configuration (3 mA anodal right IFC‐ cathodal Fp1) showed limited clinical efficacy for reducing OCD symptoms and improving response inhibition. The excessive heterogeneity in OCD pathophysiology [21, 47], clinical predictors of response [66], the impact of stimulation parameters (including intensity and target region), and OCD subtypes [67] are contributing factors to NIBS treatment response in OCD that could affect the limited efficacy of our intervention.

### 4.4 Limitations, Strengths, and Future Directions

Limitations include the absence of neuroimaging and/or neurophysiological data that could have provided more information about the neurophysiological effects of the intervention and potential biomarkers of tDCS response [52, 68] across groups. Another limitation is that the trial did not include a standard active comparator arm (e.g., 2 mA). Therefore, although the protocol was motivated by previous dose‐comparison findings in OCD, the present study does not allow a definitive test of whether the observed outcomes were specific to the 3 mA intensity. Lastly, subject‐specific electric field modeling and correlation analyses were not possible. Future studies combining individualized MRI‐based electric field modeling with treatment outcomes could clarify interindividual variability in response. The strengths of our study were careful monitoring of symptom and cognitive performance changes across 5 time‐points, and assessment of cognitive functions in addition to clinical assessment. Future larger, multi‐site trials with titration of stimulation parameters (intensity, polarity) along with neuroimaging data could clarify the mechanisms of effects for right IFC tDCS for OCD treatment.

## 5 Conclusion

To conclude, this sham‐controlled RCT found no evidence that 3 mA intensified tDCS over the right IFC outperforms sham stimulation for OCD treatment. Strong placebo effects, as seen in other tDCS studies, insufficient or ineffective induced excitability alteration in the target region, or suboptimal stimulation parameters likely contributed to the lack of differential outcomes across groups. Further tDCS studies in OCD with systematic titration of stimulation parameters and target location are needed.

## Author Contributions

**A.F.‐C.:** investigation, formal analysis (support), data curation, validation. **M.D.:** supervision, resources, project administration, validation. **A.M. and S.L.:** software (cognitive assessment). **A.G**. and **A.F.:** project administration (patient diagnosis and allocation). **R**.**S**.: Visualization (computational modeling). **M** **.A.N.:** supervision, writing – review and editing. **Z.V.:** formal analysis (lead), visualization, writing – original draft (support). **M.A.S.:** conceptualization, methodology, supervision, visualization, writing – original draft (lead), writing – review and editing.

## Funding

The authors have nothing to report.

## Ethics Statement

This study was conducted as a registered clinical trial (IRCT20230216057437N1) and received approval from the Ethics Committee of Zanjan University of Medical Sciences (Ethics code: IR.ZUMS.REC.1401.339). All procedures were performed in accordance with the ethical standards of the institutional ethics committee and the Declaration of Helsinki.

## Conflicts of Interest

Michael A. Nitsche is a member of the Scientific Advisory Boards of Neuroelectrics and Precisis. The other authors declare no conflicts of interest. Dr. Ricardo Salvador works for Neuroelectrics Barcelona SLU, a company developing computationally driven brain stimulation solutions.

## Supporting information


**Table S1:** Means and SDs of outcome measures.
**Table S2:** Means and SDs of obsessive‐compulsive symptoms.
**Table S3:** Mean and SD and ANOVA results for reported side effects.

**Data S1:** CONSORT 2010 checklist of information to include when reporting a randomized trial.

## Data Availability

The data that support the findings of this study are available on request from the corresponding authors. The data are not publicly available due to privacy or ethical restrictions.
