# Supplementary material for: Limited Efficacy of 3 mA Intensified tDCS of the Right Inferior Frontal Cortex for OCD Treatment: A Randomized, Double‐Blind, Sham‐Controlled Study
Source: CNS Neurosci Ther. 2026 May 24;32(5):e70927. doi: 10.1002/cns.70927 (PMC13239271; doi:10.1002/cns.70927)
Supplement: Supplementary file 1 — Table S1: Means and SDs of outcome measures. Table S2: Means and SDs of obsessive‐compulsive symptoms. Table S3: Mean and SD and ANOVA results for reported side effects. [file CNS-32-e70927-s001.docx]

***Clinical and cognitive measures***

***Yale-Brown Obsessive-Compulsive Scale (Y-BOCS)***

The Y-BOCS (Goodman et al., 1989a; Goodman et al., 1989b) is a reliable semi-structured interview conducted by clinicians that evaluates the presence and intensity of OCD symptoms experienced in the past week (Benito and Storch, 2011). Although the Y-BOCS includes a comprehensive Symptom Checklist and a Severity scale, we concentrated solely on the Severity scale. This scale comprises a total score and subscales that measure the severity of obsessions and compulsions, respectively. It consists of ten questions, with scores from 0 to 4 (with 4 indicating the most severe), which assess factors such as time spent, interference, distress, resistance, and control over obsessions and compulsions, resulting in a total score that ranges from 0 to 40. In the present study, internal consistency using Cronbach's alpha method for this questionnaire was obtained as 0.854.

***The Beck Anxiety Inventory (BAI)***

The BAI (Beck et al., 1988) is a self-report questionnaire consisting of 21 items, prompting participants to evaluate how much anxiety symptoms have affected them in the past week. Respondents rate each item on a scale from 0 (not at all) to 3 (severely). The total score can range from 0 to 63, with higher scores reflecting greater anxiety symptoms. In the study by Beck et al. (1988), the BAI demonstrated strong reliability and validity (Beck et al., 1988). In the present study, internal consistency using Cronbach's alpha method for this questionnaire was obtained as 0.924.

***The Beck Depression Inventory-II (BDI-II)***

BDI-II is based on the DSM-IV diagnostic criteria for depression. It consists of 21 questions, with responses rated on a scale from 0 to 3. Higher scores reflect greater depressive symptoms. Specifically, a score of 0 to 13 reflects minimal depression, 14 to 19 indicates mild depression, 20 to 28 signifies moderate depression, and scores of 29 to 63 are classified as severe depression (Beck et al., 1996). In the present study, internal consistency using Cronbach's alpha method for this questionnaire was obtained as 0.906.

- - 1. *Cognitive* ***assessment***

The Cambridge Neuropsychological Test Automated Battery (CANTAB [Cognitive assessment software], 2018) is a computerized collection of cognitive assessments that were used in this study to assess the following areas: cognitive flexibility (assessed through the Intra-Extra Dimensional Set Shift task), response inhibition (evaluated via the Stop Signal Task), and sustained attention (assessed using the Rapid Visual Information Processing task. The CANTAB tests have been thoroughly detailed elsewhere (Sahakian and Owen, 1992; Langley et al., 2023).

***The Rapid Visual Processing task***

RVP evaluates sustained visual attention. A white square is displayed in the center of the computer screen, within which digits ranging from 2 to 9 appear in a pseudo-random sequence at a speed of 100 digits per minute. Participants are asked to identify specific target sequences of digits (such as 2-4-6 or 3-5-7) and record their responses using a press pad. Target sequences are presented at a rate of 16 every 2 minutes, and they remain visible on the screen throughout the trial. The outcome measures of performance were RVP-A score, total hits, and latency.

***Intra-Extra Dimensional Set Shift task***

The IED test evaluates the ability to acquire and reverse rules related to visual discrimination and attentional set-shifting. Participants in the study are instructed to select the correct figure from two artificial dimensions (form and color) and use feedback to identify the correct stimulus. After providing six correct responses, the rule and/or stimuli would change. Initially, participants could easily differentiate the visual stimuli based on one relevant dimension, leading to intra-dimensional shifts in the rules. Later, the stimuli could only be distinguished through a combination of both dimensions, resulting in extra-dimensional shifts. The task consisted of nine stages, with intra-dimensional and extra-dimensional rule changes occurring at stages 6 and 8, respectively. The dependent variables included latency and stages completed.

***The Stop Signal Task***

SST assesses response inhibition. Participants see left and right arrows displayed randomly on a screen and are required to press the corresponding button as quickly as possible during the go trials. However, they must refrain from pressing a button if they hear an auditory signal, which follows the appearance of an arrow (no-go trial). The outcome measures include accuracy, reaction time and the proportion of successful stops.

Table S1. Means and SDs of outcome measures

| Measure | Outcome variable | Time | Active tDCS | Sham tDCS | *p*-value |
| --- | --- | --- | --- | --- | --- |
|  |  |  | M (SD) | M (SD) |  |
| Y-BOCS | Score | Pre-intervention | 28.25 (7.16) | 26.70 (5.06) | 0.435 |
|  |  | Post-intervention | 22.50 (9.61) | 22.55 (5.37) |  |
|  |  | 2-week follow-up | 22.12 (10.68) | 21.25 (6.78) |  |
|  |  | 4-week follow-up | 23.70 (9.26) | 21.85 (7.65) |  |
|  |  | 3-month follow-up | 22.49 (9.68) | 21.30 (6.14) |  |
| BDI-II | Score | Pre-intervention | 30.70 (15.52) | 23.40 (10.26) | 0.087 |
|  |  | Post-intervention | 21.65 (15.56) | 18.90 (10.30) |  |
|  |  | 2-week follow-up | 24.60 (17.81) | 18.05 (10.22) |  |
|  |  | 4-week follow-up | 24.96 (15.45) | 19.70 (10.36) |  |
|  |  | 3-month follow-up | 23.34 (17.00) | 18.91 (9.47) |  |
| BAI | Score | Pre-intervention | 28.50 (11.83) | 21.45 (14.35) | 0.098 |
|  |  | Post-intervention | 19.20 (12.66) | 14.80 (10.52) |  |
|  |  | 2-week follow-up | 19.35 (12.83) | 15.45 (11.65) |  |
|  |  | 4-week follow-up | 19.57 (12.23) | 15.90 (10.52) |  |
|  |  | 3-month follow-up | 19.02 (14.77) | 16.13 (11.24) |  |
| IED | Latency | Pre-intervention | 186014.40 (74938.95) | 161228.70 (51688.55) | 0.231 |
|  |  | Post-intervention | 156828.75 (75070.52) | 126213.45 (59405.02) |  |
|  |  | 2-week follow-up | 116881.25 (39538.44) | 109654.80 (60753.56) |  |
|  |  | 4-week follow-up | 110412.85 (49670.54) | 109907.00 (59257.18) |  |
|  |  | 3-month follow-up | 116936.29 (44719.90) | 119315.54 (79847.82) |  |
|  | Stages completed | Pre-intervention | 7.90 (1.07) | 8.35 (0.93) | 0.165 |
|  |  | Post-intervention | 8.15 (0.99) | 8.45 (0.89) |  |
|  |  | 2-week follow-up | 8.28 (1.01) | 8.65 (0.75) |  |
|  |  | 4-week follow-up | 8.40 (0.94) | 8.60 (0.82) |  |
|  |  | 3-month follow-up | 8.26 (1.24) | 8.77 (0.65) |  |
| SST | Reaction Time | Pre-intervention | 353.58 (91.73) | 346.14 (84.48) | 0.791 |
|  |  | Post-intervention | 335.70 (74.08) | 339.97 (73.97) |  |
|  |  | 2-week follow-up | 345.53 (79.39) | 321.32 (77.52) |  |
|  |  | 4-week follow-up | 354.74 (85.78) | 331.26 (57.09) |  |
|  |  | 3-month follow-up | 368.06 (84.53) | 355.36 (115.32) |  |
|  | Proportion of  successful stops | Pre-intervention | 0.53 (0.17) | 0.57 (0.14) | 0.443 |
|  |  | Post-intervention | 0.55 (0.14) | 0.55 (0.13) |  |
|  |  | 2-week follow-up | 0.52 (0.19) | 0.55 (0.14) |  |
|  |  | 4-week follow-up | 0.52 (0.16) | 0.54 (0.14) |  |
|  |  | 3-month follow-up | 0.54 (0.15) | 0.58 (0.12) |  |
|  | SST Accuracy | Pre-intervention | 283.30 (13.93) | 287.40 (10.77) | 0.304 |
|  |  | Post-intervention | 282.50 (14.56) | 286.80 (11.79) |  |
|  |  | 2-week follow-up | 280.92 (15.94) | 286.90 (11.75) |  |
|  |  | 4-week follow-up | 281.28 (17.21) | 285.90 (11.20) |  |
|  |  | 3-month follow-up | 281.44 (16.88) | 286.24 (13.98) |  |
| RVP | RVP A | Pre-intervention | 0.86 (0.06) | 0.87 (0.05) | 0.477 |
|  |  | Post-intervention | 0.88 (0.06) | 0.89 (0.06) |  |
|  |  | 2-week follow-up | 0.90 (0.06) | 0.89 (0.06) |  |
|  |  | 4-week follow-up | 0.90 (0.07) | 0.90 (0.06) |  |
|  |  | 3-month follow-up | 0.89 (0.06) | 0.91 (0.05) |  |
|  | RVP Hits | Pre-intervention | 13.90 (4.92) | 14.05 (5.26) | 0.926 |
|  |  | Post-intervention | 16.45 (4.62) | 15.90 (5.49) |  |
|  |  | 2-week follow-up | 18.01 (5.35) | 16.50 (5.75) |  |
|  |  | 4-week follow-up | 16.70 (6.18) | 17.15 (5.84) |  |
|  |  | 3-month follow-up | 17.19 (4.81) | 18.21 (5.32) |  |
|  | RVP Latency | Pre-intervention | 621.18(172.56) | 572.32(125.62) | 0.312 |
|  |  | Post-intervention | 533.91(158.26) | 528.28(117.68) |  |
|  |  | 2-week follow-up | 522.28(172.60) | 516.69(77.12) |  |
|  |  | 4-week follow-up | 536.12(158.99) | 498.55(77.69) |  |
|  |  | 3-month follow-up | 508.30(147.65) | 521.05(120.93) |  |

tDCS = transcranial Direct Current Stimulation; M = Mean; SD = Standard Deviation; Y-BOCS = Yale-Brown Obsessive-Compulsive Scale; BAI = Beck Anxiety Inventory; BDI-II = Beck Depression Inventory-II; RVP = Rapid Visual Processing; IED = Intra-Extra Dimensional Set Shift; SST = Stop Signal Task; *Note*: *p* values refer to baseline (pre-intervention) measurement comparisons using ANOVA tests

Table S2: Means and SDs of obsessive-compulsive symptoms

| **Symptom** | Active tDCS (n = 20) | | | | | Sham tDCS (n = 20) | | | | |
| --- | --- | --- | --- | --- | --- | --- | --- | --- | --- | --- |
|  | Pre | Post | 2-w | 4-w | 3-m | Pre | Post | 2-w | 4-w | 3-m |
| Obsession | 14.35 (3.57) | 11.75 (4.41) | 11.40 (4.98) | 12.25 (4.48) | 11.34 (4.58) | 13.30 (2.25) | 11.15 (3.01) | 10.45 (4.06) | 10.75 (3.82) | 9.85 (2.85) |
| Compulsion | 13.90 (3.93) | 10.75 (5.55) | 10.72 (6.23) | 11.45 (5.27) | 11.15 (5.34) | 13.40 (3.20) | 11.40 (3.02) | 10.80 (3.22) | 11.10 (4.22) | 11.45 (4.15) |

Values are presented as means (standard deviations). *Note*: tDCS = transcranial Direct Current Stimulation; M = Mean; SD = Standard Deviation.Pre = pre-intervention; post = post-intervention; 2w = 2-week follow up; 4w = 4-week follow up; 3-m = 3-month follow up.

Table S3: Mean and SD and ANOVA results for reported side effects

| **Reported side effect** | tDCS groups | | *F*(*p*-value) |
| --- | --- | --- | --- |
|  | Active (*n* = 20) | Sham (n = 20) |  |
| Itching | 4.65 (1.14) | 3.20 (0.89) | 20.099 (<0.001) |
| Burning | 5.25 (1.21) | 3.05 (0.76) | 47.525 (<0.001) |
| Pain | 4.80 (0.83) | 3.85 (0.88) | 12.359 (0.001) |
| Skin redness | 5.00 (1.34) | 2.80 (1.28) | 28.209 (<0.001) |
| Trouble concentration | 3.20 (0.70) | 3.30 (1.13) | 0.114 (0.738) |

Values are presented as means (standard deviations). *Note*: each value represents the average of side effects averaged across all 20 tDCS sessions.

**References**

Beck AT, Steer RA, Brown GK (1996) Beck depression inventory.

Beck AT, Epstein N, Brown G, Steer RA (1988) An inventory for measuring clinical anxiety: psychometric properties. Journal of consulting and clinical psychology 56:893.

Benito K, Storch EA (2011) Assessment of obsessive–compulsive disorder: review and future directions. Expert Review of Neurotherapeutics 11:287-298.

Goodman WK, Price LH, Rasmussen SA, Mazure C, Delgado P, Heninger GR, Charney DS (1989a) The yale-brown obsessive compulsive scale: II. Validity. Archives of general psychiatry 46:1012-1016.

Goodman WK, Price LH, Rasmussen SA, Mazure C, Fleischmann RL, Hill CL, Heninger GR, Charney DS (1989b) The Yale-Brown obsessive compulsive scale: I. Development, use, and reliability. Archives of general psychiatry 46:1006-1011.

Langley C, Sahakian BJ, Robbins TW (2023) Cambridge Neuropsychological Test Automated Battery (CANTAB). The SAGE Handbook of Clinical Neuropsychology: Clinical Neuropsychological Assessment and Diagnosis:435.

Sahakian BJ, Owen A (1992) Computerized assessment in neuropsychiatry using CANTAB: discussion paper. Journal of the Royal Society of medicine 85:399.
